# Tripartite strategy for dual reduction of radiation and iodine dose in obese CCTA: High-iodine contrast, 80 kVp, and deep learning reconstruction

**DOI:** 10.1097/MD.0000000000045725

**Published:** 2025-10-31

**Authors:** Qiuhua Zhang, Ai’zhu Sheng, Hong Ren, Kun Wang

**Affiliations:** a Health Information Department, Sir Run Run Shaw Hospital, Zhejiang University School of Medicine, Hangzhou, China; b Department of Radiology, Ningbo No.2 Hospital, Ningbo, China; c Department of Radiology, Sir Run Run Shaw Hospital, Zhejiang University School of Medicine, Hangzhou, China.

**Keywords:** coronary computed tomography angiography, deep learning image reconstruction, high-iodine-concentration contrast media, obese, tripartite protocol

## Abstract

Coronary CT angiography (CCTA) in obese patients is challenging due to high radiation exposure, elevated iodine load, and increased image noise. We evaluated a tripartite low-dose strategy combining high-iodine-concentration contrast media (HCM, 400 mgI/mL), 80-kVp scanning, and deep learning image reconstruction (DLIR) in obese patients (BMI ≥30 kg/m^2^) to simultaneously reduce radiation and iodine dose while maintaining diagnostic quality. In this prospective, single-center trial, 100 obese patients were randomized to either a low-dose group (80 kVp, HCM at 0.6 mL/kg, DLIR reconstruction; n = 50) or a standard-dose group (120 kVp, 350 mgI/mL contrast at 0.9 mL/kg, adaptive statistical iterative reconstruction [ASiR]-V 50% reconstruction; n = 50). Low-dose scans were also reconstructed with ASiR-V 50% for internal comparison. Objective (noise, signal-to-noise ratio, contrast-to-noise ratio) and subjective (5-point scale by blinded radiologists) image quality, radiation dose, total iodine load, and diagnostic performance (vs invasive coronary angiography in a 20-patient subgroup) for detecting ≥50% stenosis were compared. The low-dose protocol significantly reduced effective radiation dose by 48.6% (1.06 ± 0.26 mSv vs 2.27 ± 0.42 mSv; *P* <.001) and total iodine load by 23.8% (19.2 ± 2.1 gI vs 25.2 ± 2.4 gI; *P* <.001) compared to standard protocol. DLIR at 80-kVp significantly reduced image noise (aortic root: 16.5 ± 3.0 HU vs 25.1 ± 4.0 HU for ASiR-V at 80 kVp; *P* <.001) and improved contrast-to-noise ratio (e.g., proximal left anterior descending: +41.6%, *P* <.001) and signal-to-noise ratio (e.g., distal right coronary artery: +63.8%, *P* <.001). Subjective image quality scores were significantly higher for DLIR (mean 4.4 ± 0.5) versus low-dose ASiR-V (3.0 ± 0.7) and standard-dose ASiR-V (4.1 ± 0.7; *P* <.001), with excellent inter-observer agreement (κ = 0.88). 94% of DLIR datasets received optimal noise scores (≥4/5) versus 0% for low-dose ASiR-V. Against invasive coronary angiography, the low-dose protocol achieved 100% patient-level sensitivity and negative predictive value, with 91.7% vessel-level accuracy. Combining HCM, 80-kVp scanning, and DLIR significantly reduces radiation exposure and iodine load in obese CCTA patients without compromising image quality or diagnostic accuracy compared to standard protocols, offering a safer alternative, particularly for patients with renal impairment.

## 1. Introduction

Coronary CT angiography (CCTA) serves as the primary diagnostic modality for coronary artery disease (CAD).^[1–3]^ Yet, its application in obese patients (BMI ≥30 kg/m^2^) remains challenging due to increased radiation exposure, higher risks of contrast-induced nephropathy (CIN) from larger contrast volumes, and degraded image quality from elevated image noise.^[4]^ Conventional protocols for obese patients typically employ high tube voltage (120–140 kV) and standard iodine concentration contrast media (300–350 mgI/mL) to ensure sufficient photon penetration, but this approach inevitably raises radiation dose (effective dose >5 mSv) and contrast volume (>60 mL), particularly in patients with borderline renal function (eGFR 45–60 mL/min/1.73m²).^[5]^

Recent advances propose combining low-tube-voltage (80–100 kV) with high-iodine-concentration contrast media (HCM, 370–400 mgI/mL) to leverage the iodine’s K-edge effect for improved contrast-to-noise ratio (CNR) at reduced radiation dose.^[6]^ However, the inherent photon starvation in obese patients under low-kV protocols often amplifies noise, particularly in distal coronary segments, limiting diagnostic confidence.^[7]^ Deep learning-based image reconstruction (DLIR) algorithms, trained on extensive clinical datasets, offer a potential solution by suppressing noise while preserving vascular details.^[8,9]^

Despite promising results in non-obese cohorts, the synergistic efficacy of HCM, low-kV, and DLIR specifically in obese populations (BMI ≥30 kg/m²) for simultaneously optimizing radiation dose, iodine load, and diagnostic image quality remains underexplored. Prior studies focusing on dose reduction in obesity have rarely achieved substantial reductions in both radiation and iodine load concurrently,^[7]^ and the application of advanced DLIR at very low kVp (80 kVp) in this population is limited. This study aimed to validate whether this tripartite strategy can simultaneously reduce radiation and iodine doses while maintaining image quality and diagnostic accuracy comparable to conventional protocols and invasive coronary angiography (ICA).

## 2. Materials and methods

### 2.1. Patient COHORT

**Figure 1.**
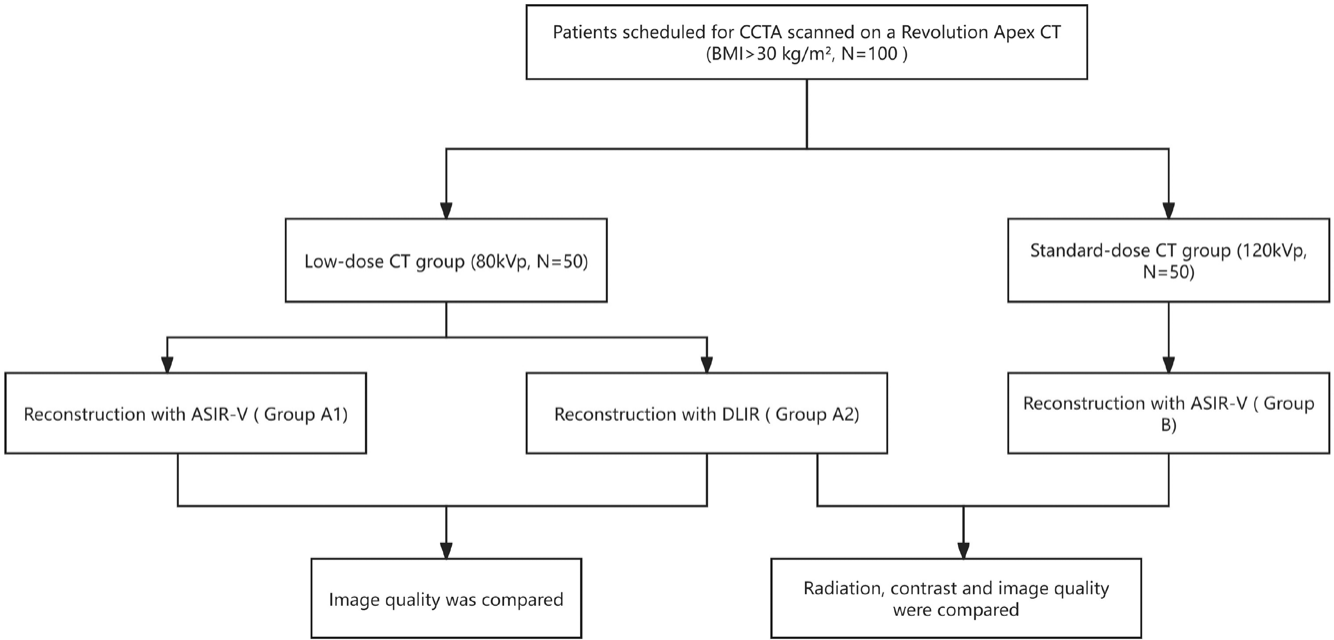
Flowchart of the study.

This prospective, single-center study was approved by the Institutional Review Board of Sir Run Run Shaw Hospital, Zhejiang University School of Medicine (Approval Number: K2024473), and written informed consent was provided by all participants. Enrollment of 100 obese patients (BMI ≥30 kg/m^2^) presenting with suspected CAD took place between March 2024 and April 2025. Randomization assigned participants to either an experimental low-dose group (n = 50) or a standard-dose control group (n = 50) (Fig. 1). Exclusion criteria comprised: BMI < 30 kg/m^2^, pregnancy, age <18 years, contraindications to iodinated contrast media (including allergy or renal insufficiency defined as serum creatinine > 120 μmol/L or eGFR <60 mL/min/1.73m^2^), and a history of heart failure, prior coronary stent implantation, or cardiac surgery.

### 2.2. Imaging acquisition

All scans were performed on a 256-slice Revolution Apex Expert CT scanner (GE Healthcare) using a prospective ECG-triggered axial mode. The experimental group underwent CCTA at 80 kVp with high-iodine-concentration contrast media (Iomeron 400, 400 mgI/mL; 0.6 mL/kg over 10 seconds, Group A), while the control group was scanned at 120 kVp with standard contrast (350 mgI/mL; 0.9 mL/kg over 10 seconds, Group B). The injection flow rate was capped at 6.5 mL/s using a pre-warmed contrast agent and a dual-syringe injector. automated tube current modulation was applied with a noise index of 30 HU for the experimental group and 20 HU for the control group, selected based on institutional experience and preliminary testing to balance dose and anticipated noise levels for each reconstruction type. The scan range (120–160 mm) and collimation (0.625 mm) were adjusted based on individual heart size. The CT scanner employed auto gating to automatically determine optimal cardiac acquisition phases based on pre-scan heart rate (HR) measurements. For patients with HR <66 bpm, the exposure window was set at 70% to 80% of the RR interval; for those with HR between 66 and 85 bpm, dual-phase acquisition at 40% to 55% and 70% to 80% RR intervals was used; whereas for HR >86 bpm, the 40% to 55% RR interval was selected. Patients exhibiting HR variability exceeding 10 beats/min underwent whole-heart imaging captured within a single cardiac cycle. Cardiac motion correction was uniformly applied during reconstruction using the SnapShot Freeze 2 algorithm (GE Healthcare) for both groups. Reconstruction protocols differed by cohort: experimental group scans (80 kVp) were processed with both adaptive statistical iterative reconstruction (ASiR-V 50%, designated A1) and DLIR (TrueFidelity™, GE Healthcare; High-strength, A2), while control group scans (120 kVp) utilized ASiR-V 50% (B). All reconstructed images employed identical parameters of 0.625 mm slice thickness and 0.625 mm interval for subsequent analysis and diagnostic evaluation.

### 2.3. Image quality analysis and evaluation

Quantitative measurements included CT values and standard deviations (SDs) for the luminal segment of 7 anatomical regions: aortic root (AO), proximal left main coronary artery (LMCA-P), middle left anterior descending (LAD) artery (LAD-M), distal LAD artery (LAD-D), middle left circumflex artery (LCX-M), distal left circumflex artery (LCX-D), proximal right coronary artery (RCA-P), middle right coronary artery(RCA-M), distal right coronary artery (RCA-D), and perivascular adipose tissue (PVAT). Image noise was defined as the SD of AO measurements. region-of-interest (ROI) placement followed standardized protocols: a 90 mm^2^ ROI was centrally positioned within the AO lumen while circumventing vascular walls, calcifications, and plaques; all other vascular sites utilized 1 mm^2^ ROIs maximized within vessel lumens while avoiding wall contact and plaque. Signal-to-noise ratio (SNR) and CNR were calculated using the following equations:


$$\begin{array}{l} SNR=\frac{Mean\;CT\;value\;\left(ROI\right)}{Noise} \end{array}$$ (1)

$$\begin{array}{l} CNR=\frac{Mean\;CT\;value\;\left(ROI\right)-Mean\;CT\;value\;\left(PVAT\right)}{SD\;\left(PVAT\right)} \end{array}$$ (2)

Two radiologists specializing in CCTA with over a decade of diagnostic experience independently conducted blinded qualitative assessments using the American Heart Association 5-point scoring system,^[6]^ remaining unaware of scanning and reconstruction protocols. They evaluated 4 key parameters: subjective image noise, vessel attenuation, edge sharpness, and overall image quality. The scoring criteria ranged from 1 (nondiagnostic: severe noise, inadequate attenuation, non-definable vessel edges) to 5 (excellent: minimal noise, high attenuation, sharply defined edges), with intermediate scores reflecting progressively improving quality – 2 indicating poor quality with diagnostic difficulty, 3 representing acceptable diagnostic quality, and 4 denoting good quality. Studies scoring ≥3 were deemed diagnostically adequate. For coronary analysis, segments exceeding 1.5 mm diameter were evaluated per-vessel, with the lowest segment score determining the vessel-level image quality. The poorest score among the 3 major coronary vessels (right coronary artery [RCA], LAD, and left circumflex [LCX]) subsequently defined each patient’s overall image quality score. Final subjective scores were derived by averaging both radiologists’ assessments for intergroup comparisons, while the original independent scores facilitated inter-rater consistency analysis.

Two independent observers (7 and 10 years of cardiac CT experience, respectively), blinded to scan protocols, reconstruction methods, and ICA results, evaluated CCTA images according to the Society of Cardiovascular Computed Tomography (SCCT) 18-segment model.^[10,11]^ All coronary segments ≥1.5 mm diameter were included. Evaluable segments underwent independent assessment for significant stenosis, defined as ≥50% luminal diameter reduction,^[12]^ analyzed at 3 levels: per-segment, per-vessel (left main, LAD, left circumflex [LCX], RCA), and per-patient. Disagreements between observers regarding stenosis severity were resolved through consensus review during joint sessions. Segments deemed non-evaluable were classified as positive findings for diagnostic interpretation.

ICA was performed within 4 weeks following CCTA, utilizing standard Judkins technique with acquisition of ≥2 orthogonal projections per coronary vessel. All angiographic segments were evaluated by an independent interventional cardiologist blinded to CCTA findings and clinical data. Consistent with the CCTA analysis, the SCCT 18-segment model was applied, with ≥50% diameter stenosis considered diagnostic of obstructive CAD.

### 2.4. Radiation dose

For each patient, the volumetric CT dose index (CTDIvol) and dose-length product from CCTA examinations were documented. The effective radiation dose (ED) was derived using the conversion formula ED = k × dose-length product, where the tissue-weighting factor k = 0.014 mSv/(mGy·cm) as established in prior dosimetry studies.^[13]^

### 2.5. Statistics analysis

Continuous variables following normal distribution are reported as mean ± SD, while categorical data are expressed as frequencies with percentages. Group comparisons employed Student *t* test for normally distributed continuous variables and the Mann–Whitney U test for non-normally distributed data. Categorical variables were analyzed using chi-square tests, with Fisher exact test substituted when expected cell frequencies fell below 5. Inter-observer agreement for subjective scores was quantified by Cohen kappa statistic, interpreted as follows: 0 to 0.20 (slight), 0.21 to 0.40 (fair), 0.41 to 0.60 (moderate), 0.61 to 0.80 (substantial), and 0.81 to 1.00 (almost perfect). Statistical significance was defined at *P* < .05. All analyses were conducted using R software (version 3.3.1; R Foundation for Statistical Computing).

## 3. Results

### 3.1. Patient characteristics and dose parameters

A prospective cohort of 100 obese patients (BMI ≥30 kg/m^2^) was randomized into low-dose (80 kVp + DLIR, n = 50) and standard-dose (120 kVp + ASiR-V, n = 50) groups, with no significant differences in baseline demographics including age (62 ± 11 vs 61 ± 10 years, *P* = .654), gender distribution (68% vs 64% male, *P* = .732), BMI (31.2 ± 1.5 vs 30.9 ± 1.3 kg/m^2^, *P* = .311), or cardiovascular risk profiles (all *P* > .05) (Table 1). The low-dose protocol achieved a 48.6% reduction in effective radiation dose (1.06 ± 0.26 vs 2.27 ± 0.42 mSv, *P* < .001), a 23.8% decrease in total iodine load (19.2 ± 2.1 vs 25.2 ± 2.4 gI, *P* < .001), and a hemodynamically safer contrast injection flow rate (4.8 ± 0.5 vs 6.3 ± 0.4 mL/s, *P* < .001) while maintaining diagnostic efficacy.

**Table 1:** Demographic, clinical, and radiation dose characteristics.

Parameter	Low-dose group (n = 50)	Standard-dose group (n = 50)	*P*-value
Age, years	62 ± 11	61 ± 10	.654
Gender (male/female)	34/16	32/18	.732
BMI, kg/m^2^	31.2 ± 1.5	30.9 ± 1.3	.311
Heart rate, bpm	70.5 ± 12.3	68.9 ± 13.8	.532
Arrhythmia, n (%)	5 (10.0)	4 (8.0)	.705
Cardiovascular risk factors			
Hypertension, n (%)	24 (48.0)	26 (52.0)	.689
Dyslipidemia, n (%)	18 (36.0)	22 (44.0)	.409
Diabetes, n (%)	15 (30.0)	20 (40.0)	.264
Smoking, n (%)	28 (56.0)	25 (50.0)	.549
Family history of CAD, n (%)	26 (52.0)	34 (68.0)	.088
Referral reason, n (%)			
Physical examination	5 (10.0)	4 (8.0)	–
CAD-related symptoms	24 (48.0)	32 (64.0)	–
Non-CAD symptoms	12 (24.0)	4 (8.0)	–
Pre-surgical screening	9 (18.0)	10 (20.0)	–
Stenosis severity, n (%)			
No stenosis	22 (44.0)	21 (42.0)	–
<50% stenosis	15 (30.0)	15 (30.0)	–
≥50% stenosis	13 (26.0)	14 (28.0)	–
Myocardial bridge, n (%)	5 (10.0)	3 (6.0)	0.712
Radiation and contrast dose			
Contrast flow rate (mL/s)	4.8 ± 0.5	6.3 ± 0.4	<.001
Contrast volume (mL)	48.0 ± 5.2	72.0 ± 6.8	<.001
Total iodine load (gI)	19.2 ± 2.1	25.2 ± 2.4	<.001
CTDIvol (mGy)	5.2 ± 1.3	10.8 ± 2.0	<.001
DLP (mGy·cm)	75.6 ± 18.4	162.0 ± 30.2	<.001
Effective dose (mSv)	1.06 ± 0.26	2.27 ± 0.42	<.001

BMI = body mass index, CAD = coronary artery disease, CTDIvol = volumetric CT dose index, DLP, dose-length product, CTDIvol = volume CT dose index, DLP = dose-length-product, ED = effective dose, ED = effective dose, mSv = millisieverts.

### 3.2. Image quality assessment

Objective analysis of identical 80-kVp scans revealed that DLIR reconstruction (A2) significantly reduced image noise compared to ASiR-V (A1), with aortic root noise decreasing from 25.1 ± 4.0 HU to 16.5 ± 3.0 HU (*P* <.001), while maintaining equivalent CT attenuation values across all coronary segments (all *P* >.05) (Table 2). This noise suppression resulted in significant improvements in vessel conspicuity: DLIR increased CNR by 41.6% in the proximal LAD (18.2 ± 3.1 vs 12.5 ± 2.8, *P* <.001) and SNR by 63.8% in the distal RCA (26.2 ± 4.2 vs 16.0 ± 2.8, *P* <.001). Compared to the standard-dose control (120 kVp + ASiR-V), DLIR delivered superior small-vessel visualization, evidenced by a 19.7% increase in CNR in the distal LCX (13.2 ± 2.4 vs 11.0 ± 2.2, *P* = .02) and significantly higher SNR and CNR values in most coronary segments (Table 2).

**Table 2:** Results of the objective and subjective quality analysis.

	Group A1 (80 kVp + ASiR-V)	Group A2 (80 kVp + DLIR)	Group B (120 kVp + ASiR-V)	A2 versus A1 *P*-value	*A2* versus *B P*-value
CT values (HU)					
AO	480 ± 55	482 ± 54	465 ± 48	.85	0.07
LMCA-P	480 ± 52	482 ± 51	465 ± 45	.82	0.06
LAD-M	465 ± 50	467 ± 49	450 ± 42	.80	0.04
LAD-D	445 ± 55	447 ± 54	430 ± 48	.79	0.10
LCX-M	455 ± 51	457 ± 50	440 ± 46	.81	0.08
LCX-D	435 ± 53	437 ± 52	425 ± 47	.83	0.12
RCA-P	495 ± 54	497 ± 53	480 ± 48	.86	0.07
RCA-M	470 ± 52	472 ± 51	455 ± 45	.82	0.04
RCA-D	440 ± 55	442 ± 54	430 ± 48	.80	0.12
PVAT	-86.8 ± 11.5	-86.5 ± 11.2	-85.5 ± 11.2	.78	0.42
SNR values					
AO	19.1 ± 3.8	29.2 ± 5.3	23.3 ± 4.4	<.001	<0.001
LMCA-P	18.0 ± 3.5	28.7 ± 5.2	23.1 ± 4.3	<.001	<0.001
LAD-M	17.6 ± 3.2	27.9 ± 4.8	21.9 ± 3.9	<.001	0.003
LAD-D	16.8 ± 3.0	26.8 ± 4.5	20.5 ± 3.7	<.001	0.005
LCX-M	17.3 ± 3.1	27.6 ± 4.7	22.1 ± 3.8	<.001	0.004
LCX-D	16.2 ± 2.9	26.4 ± 4.3	19.8 ± 3.6	<.001	0.006
RCA-P	18.7 ± 3.6	29.6 ± 5.4	24.0 ± 4.5	<.001	0.001
RCA-M	17.5 ± 3.3	28.2 ± 4.9	22.3 ± 4.0	<.001	0.003
RCA-D	16.0 ± 2.8	26.2 ± 4.2	19.6 ± 3.5	<.001	0.007
CNR values					
AO	24.8 ± 5.1	35.1 ± 6.5	28.5 ± 5.2	<.001	<0.001
LMCA-P	12.5 ± 2.8	18.2 ± 3.1	15.3 ± 2.9	<.001	0.001
LAD-M	11.9 ± 2.5	16.8 ± 2.9	13.7 ± 2.6	<.001	0.01
LAD-D	10.2 ± 2.3	14.5 ± 2.7	12.1 ± 2.4	<.001	0.03
LCX-M	12.3 ± 2.4	16.9 ± 2.8	14.2 ± 2.5	<.001	0.005
LCX-D	9.5 ± 2.0	13.2 ± 2.4	11.0 ± 2.2	<.001	0.006
RCA-P	14.2 ± 2.9	19.0 ± 3.3	16.1 ± 2.8	<.001	0.003
RCA-M	12.0 ± 2.6	16.5 ± 2.9	14.0 ± 2.6	<.001	0.01
RCA-D	9.8 ± 2.1	13.9 ± 2.5	11.2 ± 2.3	<.001	0.02
Image noise	25.1 ± 4.0	16.5 ± 3.0	20.0 ± 3.7	<.001	<0.001

AO = aortic root, LAD-D = distal left anterior descending, LAD-M = middle left anterior descending, LCX-D = distal left circumflex, LCX-M = middle left circumflex, LMCA-P = proximal left main coronary artery, PVAT = perivascular adipose tissue, RCA-D = distal coronary right artery, RCA-M = middle coronary right artery, RCA-P = proximal right coronary artery.

Subjectively, 2 blinded radiologists rated DLIR images (A2) significantly higher than both ASiR-V reconstructions at 80 kVp (A1) (mean overall score 4.4 ± 0.5 vs 3.0 ± 0.7, *P* < .001) and the standard-dose ASiR-V reconstructions (B) (4.4 ± 0.5 vs 4.1 ± 0.7, *P* < .001) (Table 3). 94% of DLIR datasets (A2) received optimal noise scores (≥4/5) compared to 0% for A1 and 76% for B (Table 3). Inter-observer agreement for DLIR was nearly perfect (κ = 0.88), outperforming ASiR-V (κ = 0.67–0.76) and confirming consistent diagnostic confidence among reviewers.

**Table 3:** Subjective image quality (distribution) comparison.

Quality score	Low-dose group with ASiR-V (Group A1)		Low-dose group with DLIR (Group A2)		Standard-dose group with ASiR-V (Group B)	
	Reviewer 1	Reviewer 2	Reviewer 1	Reviewer 2	Reviewer 1	Reviewer 2
Image noise						
1	0	1	0	0	0	0
2	23	23	0	0	0	0
3	27	25	3	3	8	7
4	0	1	25	23	31	32
5	0	5	22	24	11	11
*K*appa value	0.66 (0.52–0.80)		0.87 (0.85–0.95)		0.73 (0.60–0.86)	
Vessel attenuation						
1	0	0	0	0	0	0
2	0	0	0	0	0	0
3	2	2	2	2	1	6
4	12	12	6	9	10	4
5	36	36	42	39	39	40
*K*appa value	0.70 (0.58–0.82)		0.85 (0.80–0.90)		0.82 (0.75–0.89)	
Overall image quality						
1	0	1	0	0	0	0
2	23	23	0	0	10	12
3	27	25	5	5	12	12
4	0	1	19	19	28	26
5	0	0	26	26	0	0
*K*appa value	0.67 (0.55–0.79)		0.88 (0.85–0.95)		0.76 (0.65–0.87)	

ASiR = adaptive statistical iterative reconstruction, DLIR = deep learning image reconstruction.

The number of non-evaluable coronary segments was low overall. In the low-dose group reconstructed with DLIR (A2), 18 segments (1.8%) out of a total 1000 segments evaluated were deemed non-evaluable. In the standard-dose group (B), 15 segments (1.5%) were non-evaluable (*P* = .68). The majority of non-evaluable segments in both groups were located in distal branches (e.g., distal RCA posterolateral segments, distal LAD), primarily attributed to motion artifacts (62% of cases) or severe calcification (31% of cases), rather than excessive image noise in the A2 group.

### 3.3. Diagnostic performance

In the 20-patient validation cohort undergoing ICA, the 80-kVp DLIR protocol (A2) demonstrated robust diagnostic performance across all levels of analysis. At the patient level (n = 20), A2 achieved perfect sensitivity (100%, 95% CI: 81.5%–100%) and negative predictive value (100%, 47.8%–100%) for detecting hemodynamically significant CAD (≥50% stenosis), with specificity reaching 85.7% (42.1%–99.6%), where the single false positive was attributed to severe calcification in an LCX segment (Agatston score 580). Vessel-level analysis (60 vessels) validated high accuracy (91.7%, 81.6%–97.2%), particularly for LAD artery stenosis, where sensitivity reached 96.0%. Segment-based evaluation (280 segments) confirmed reliable small-vessel assessment, with 92.3% sensitivity (85.6%–96.6%) and 95.1% specificity (90.2%–98.0%) in distal branches such as RCA posterolateral segments (Table 4), establishing DLIR’s capability to exclude clinically relevant stenosis while reducing radiation dose by 48.6%.

**Table 4:** Diagnostic performance of A2 protocol (80 kVp + DLIR) versus DSA.

Analysis level	Per-patient (n = 20)	Per-vessel (n = 60)	Per-segment (n = 280)
Sensitivity (95% CI)	100% (81.5%–100%)	93.3% (77.9%–99.2%)	92.3% (85.6%–96.6%)
Specificity (95% CI)	85.7% (42.1%–99.6%)	90.0% (73.5%–97.9%)	95.1% (90.2%–98.0%)
PPV (95% CI)	94.7% (74.0%–99.9%)	87.5% (71.0%–96.5%)	89.5% (81.3%–94.8%)
NPV (95% CI)	100% (47.8%–100%)	94.4% (81.3%–99.3%)	96.2% (91.8%–98.6%)
Accuracy (95% CI)	95.0% (75.1%–99.9%)	91.7% (81.6%–97.2%)	93.9% (90.5%–96.3%)

CI = confidence interval, DLIR = deep learning image reconstruction, DSA = digital subtraction angiography, NPV = negative predictive value, PPV = positive predictive value.

## 4. Discussion

**Figure 2.**
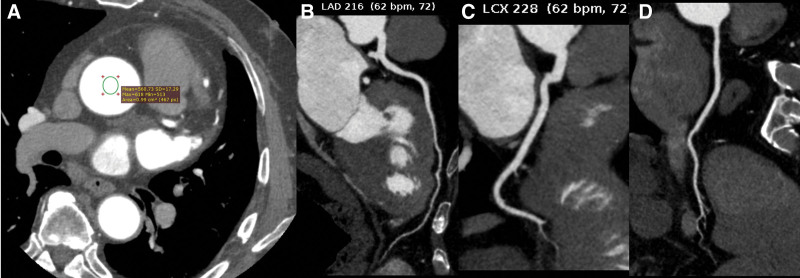
Image quality of group A2.

This study demonstrates that integrating high-iodine contrast media (HCM), low-tube-voltage (80 kVp) acquisition, and DLIR enables CCTA in obese patients (BMI ≥30 kg/m^2^) with substantially reduced radiation and iodine doses while preserving diagnostic accuracy (Fig. 2). Our tripartite protocol achieved a 48.6% reduction in effective radiation dose (*P* <.001) and a 23.8% decrease in total iodine load (*P* <.001) compared to conventional 120-kVp imaging with iterative reconstruction.^[14]^ Critically, this addresses 2 persistent limitations in obese CCTA: excessive radiation exposure (historically >5 mSv) and elevated CIN risk from high-iodine volumes.^[15]^

Previous low-dose CCTA studies have largely focused on non-obese cohorts and rarely addressed simultaneous optimization of radiation and iodine load. For instance, Wang et al achieved a 60% radiation reduction using 70 kVp + DLIR in patients with BMI <26 kg/m^2^ but did not reduce iodine load.^[16]^ In contrast, our protocol exploits the concentration advantage of HCM (400 mgI/mL) to lower contrast volume (0.6 vs 0.9 mL/kg) despite higher patient BMI (31.2 kg/m^2^). Crucially, DLIR outperformed traditional iterative reconstruction in obese patients: subjective image quality scores were significantly higher (4.4 ± 0.5 vs 3.0 ± 0.7 for ASiR-V at 80 kVp, *P* < .001), and inter-observer agreement approached perfection (κ = 0.88 vs 0.67–0.76 for ASiR-V), ensuring diagnostic reliability.

The success of our protocol hinges on complementary physical and algorithmic innovations. Low-kVp scanning leverages the photoelectric effect to amplify iodine contrast by 15% to 20% compared to 120 kVp (Table 2), while DLIR counteracts photon starvation through deep learning-based noise-structure discrimination. This dual action reduced aortic noise to 16.5 HU – 35% lower than ASiR-V at identical 80 kVp (*P* < .001) and 18% below conventional 120-kVp/ASiR-V scans. Consequently, CNR increased by 41.6% in proximal LAD and SNR by 63.8% in distal RCA versus 80 kVp + ASiR-V (both *P* <.001), outperforming Catapano et al 100-kVp DLIR results in non-obese patients.^[17]^ This mechanistic synergy explains the superiority of our protocol in small-vessel evaluation (e.g., 19.7% higher distal LCX CNR vs 120-kVp/ASiR-V group, *P* = .02), which is a critical advance for obesity imaging, where distal coronary assessment remains challenging. While Sun et al^[7]^ achieved radiation dose reduction (3.19 ± 0.96 mSv) in large patients (BMI 26–30 kg/m^2^) using a 100-kVp protocol with iterative reconstruction, this approach resulted in elevated image noise (38.53 ± 6.98 HU). In contrast, our study implemented an 80-kVp protocol for overweight patients reconstructed with DLIR. Notably, we attained both a lower radiation dose (1.06 mSv) and superior noise control (16.5 HU)–representing a 67% reduction in dose and a 57% decrease in noise compared to Sun et al Remarkably, our noise levels were even lower than the standard-dose group using state-of-the-art ASiR-V reconstruction in our cohort. Furthermore, our results in higher BMI patients surpass the CNR improvements reported by Catapano et al^[17]^ using 100-kVp DLIR in non-obese cohorts, highlighting the efficacy of the 80-kVp + HCM + DLIR synergy specifically for obesity challenges.

Diagnostic performance against invasive angiography further validates our protocol. It achieved 100% sensitivity and NPV at the patient level, with 91.7% vessel-level accuracy – surpassing prior efforts.^[10]^ While severe calcification remains a limitation (as in 1 LCX false positive), this integrated strategy establishes a new safety-efficacy benchmark for obese CCTA.

Another benefit of the 80-kVp protocol is that the required contrast media dose is greatly reduced, and the injection flow rate is consequently reduced. In our study, the contrast media volume was reduced by 33.0%, and the contrast media injection flow rate was reduced to 4.8 mL/s in the low-dose group. During CCTA scanning, the contrast media are usually injected at a higher flow rate of 5 mL/s, which may lead to vessel rupture for patients with poor vascular elasticity.^[18]^ In overweight patients, more contrast agent is usually injected, so the 80-kVp protocol can also benefit patients by reducing the flow rate and total amount of contrast agent injected. The protocol’s hemodynamic safety profile (reduced contrast flow rate: 4.8 vs 6.3 mL/s, *P* <.001) and lower iodine volume (19.2 gI vs 25.2 gI) offer critical nephroprotection for borderline renal function patients.

This single-center study (n = 100) requires validation in larger, multiethnic cohorts. While DLIR (TrueFidelity™) demonstrated exceptional noise reduction, its generalizability to other deep learning algorithms (e.g., Canon AiCE, Siemens’ SAFIRE) remains unproven. Our cohort primarily comprised Class I obese patients (mean BMI ~31 kg/m^2^); further investigation is needed in Class II (BMI ≥35 kg/m^2^) and Class III (BMI ≥40 kg/m²) obesity. Heavy calcification (Agatston >400) still challenges specificity, warranting adjunctive CT-FFR in such cases. While motion correction was applied, the impact of arrhythmias (present in ~9% of our cohort) on image quality in this protocol warrants closer examination in future studies. Finally, ICA validation was performed in a subgroup (n = 20); larger head-to-head studies are neededto confirm diagnostic accuracy. The long-term clinical impact of reduced iodine load on CIN incidence requires prospective evaluation.

## 5. Conclusion

In conclusion, this study demonstrates that the tripartite strategy of high-iodine-concentration contrast media, low-kVp scanning, and DLIR reconstruction successfully resolves the challenging “radiation-iodine dose-image quality” triad in obese patients undergoing CCTA. It achieves a substantial reduction in radiation dose (1.06 mSv) and total iodine load (19.2 gI) while preserving diagnostic accuracy comparable to conventional high-dose protocols and ICA. This approach provides a safer, high-fidelity imaging tool for coronary artery evaluation in obesity, particularly valuable for patients with renal impairment.

## Acknowledgments

This study was supported by the Medical Engineering Research Program of the National Institute of Hospital Administration, NHC (2024MEB315) and Medical Scientific Research Foundation of Zhejiang Province (2021KY291).

## Author contributions

**Conceptualization:** Kun Wang.

**Data curation:** Qiuhua Zhang, Ai’zhu Sheng, Hong Ren, Kun Wang.

**Formal analysis:** Qiuhua Zhang, Ai’zhu Sheng.

**Funding acquisition:** Ai’zhu Sheng, Hong Ren.

**Investigation:** Qiuhua Zhang, Ai’zhu Sheng, Hong Ren.

**Methodology:** Qiuhua Zhang, Ai’zhu Sheng, Kun Wang.

**Project administration:** Hong Ren, Kun Wang.

**Resources:** Qiuhua Zhang, Hong Ren.

**Software:** Qiuhua Zhang, Hong Ren.

**Supervision:** Hong Ren, Kun Wang.

**Validation:** Hong Ren, Kun Wang.

**Visualization:** Hong Ren, Kun Wang.

**Writing** – **original draft:** Qiuhua Zhang, Ai’zhu Sheng.

**Writing** – **review & editing:** Hong Ren, Kun Wang.
